# Salvianolic Acid B Attenuates Experimental Pulmonary Fibrosis through Inhibition of the TGF-β Signaling Pathway

**DOI:** 10.1038/srep27610

**Published:** 2016-06-09

**Authors:** Qingmei Liu, Haiyan Chu, Yanyun Ma, Ting Wu, Feng Qian, Xian Ren, Wenzhen Tu, Xiaodong Zhou, Li Jin, Wenyu Wu, Jiucun Wang

**Affiliations:** 1 State Key Laboratory of Genetic Engineering and Ministry of Education Key Laboratory of Contemporary Anthropology, Collaborative Innovation Center for Genetics and Development, School of Life Sciences and Institutes of Biomedical Sciences, Fudan University, Shanghai, China; 2 Shanghai Green Valley Pharmaceutical Co., Ltd, Shanghai, China; 3 Department of Dermatology, Shanghai TCM-integrated Hospital, Shanghai, China; 4 Institute of Rheumatology, Immunology and Allergy, Fudan University, Shanghai, China; 5 Division of Rheumatology, University of Texas-Houston Health Science Center, Houston, USA; 6 Division of Dermatology, Huashan Hospital, Fudan University, Shanghai, China

## Abstract

Pulmonary fibrosis is a progressive and fatal disorder. In our previous study, we found that the Yiqihuoxue formula (YQHX), a prescription of Traditional Chinese Medicine, had a curative effect on scleroderma, a typical fibrotic disease. The aim of this study was to determine the key ingredient mediating the therapeutic effects of YQHX and to examine its effect on pulmonary fibrosis, including its mechanism. Luciferase reporter assays showed that the most important anti-fibrotic component of the YQHX was *Salviae miltiorrhiza* (SM). Experiments performed using a bleomycin-instilled mouse model of pulmonary fibrosis showed that Salvianolic acid B (SAB), the major ingredient of SM, had strong anti-inflammatory and anti-fibrotic effects through its inhibition of inflammatory cell infiltration, alveolar structure disruption, and collagen deposition. Furthermore, SAB suppressed TGF-β-induced myofibroblastic differentiation of MRC-5 fibroblasts and TGF-β-mediated epithelial-to-mesenchymal transition of A549 cells by inhibiting both Smad-dependent signaling and the Smad-independent MAPK pathway. Taken together, our results suggest that SM is the key anti-fibrotic component of the YQHX and that SAB, the major ingredient of SM, alleviates experimental pulmonary fibrosis both *in vivo* and *in vitro* by inhibiting the TGF-β signaling pathway. Together, these results suggest that SAB potently inhibits pulmonary fibrosis.

Pulmonary fibrosis represents a group of devastating and largely irreversible disorders characterized by chronic inflammation and excessive deposition of collagen^1^,^2^. Pulmonary fibrosis is a very common end-stage manifestation of several diseases, including idiopathic pulmonary fibrosis (IPF), pulmonary hypertension, and scleroderma^3^,^4^,^5^.

A number of key pro-fibrotic cytokines are responsible for driving the process of fibrogenesis, including transforming growth factor-beta (TGF-β), connective tissue growth factor (CTGF), and plasminogen activator inhibitor-1 (PAI-1)^6^,^7^. Among these pro-fibrotic cytokines, TGF-β is considered one of the most potent inducers of fibroblast activation and pulmonary fibrosis pathogenesis. Binding of TGF-β to its cell surface receptors triggers intracellular signal transduction of Smad-dependent or Smad-independent pathways^8^. In the Smad-dependent pathway, Smad2 and Smad3 are phosphorylated, allowing them to complex with Smad4 and translocate from the cytoplasm into the nucleus, where they bind to a consensus Smad-binding element (SBE) on DNA. Upon binding to this element, activated Smad proteins recruit transcriptional cofactors to the targeted DNA, resulting in transcription of extracellular matrix genes like *COL1A2*, *CTGF*, and *PAI-1*^9^,^10^. TGF-β can activate several Smad-independent signaling cascades, including the MAPK pathways, some of which can regulate Smad activation^11^. In mammalian cells, there are three distinct MAPK pathways: the extracellular signal-regulated kinase (ERK) pathway, the c-Jun amino terminal kinase (JNK) pathway, and the p38 MAPK pathway. TGF-β can also induce the epithelial-to-mesenchymal transition (EMT) in alveolar epithelial cells^12^, a process that plays an important role in the formation of pulmonary fibrosis. Several lines of evidence indicate that alveolar epithelial type II cells undergo EMT to become fibroblasts during pulmonary fibrosis^13^,^14^.

Treatments for pulmonary fibrosis, such as immunosuppressants (e.g. cyclophosphamide) and antifibrotic drugs (e.g. colchicine), are commonly limited by low efficacy and severe side effects. Although two new drugs, nintedanib and pirfenidone, were recently approved by the FDA to treat idiopathic pulmonary fibrosis, neither are recommended for patients with liver problems, and pirfenidone is not recommended for patients with end-stage kidney disease or for those who require dialysis. Hence, new drugs with improved treatment efficacy and fewer side effects are still urgently needed. The use of traditional Chinese medicine (TCM) has become an increasingly attractive approach for the treatment of fibrotic disorders. In our previous study, we found that the Yiqihuoxue formula, which was provided by the Shanghai Traditional Chinese Medicine-Integrated Hospital, can attenuate dermal and pulmonary fibrosis of scleroderma in clinical applications^15^. The prescription of Yiqihuoxue formula is composed of multiple components, with two main components of *Astragalus membranaceus* and *Salvia miltiorrhiza* (SM). Salvianolic acid B (SAB) is a major ingredient of SM, and is the only commercially available monomer component extracted from SM. SAB contains seven phenolic hydroxyls and has a molecular weight of 718 (Supplementary Fig. S1). It has been reported that SAB can effectively reverse liver fibrosis in patients suffering from chronic hepatitis B^16^.

In this study, we found that SM is the most important component of Yiqihuoxue formula in anti-fibrosis. Furthermore, we examined the anti-fibrotic effect and mechanism of SAB, and demonstrated for the first time that SAB alleviated experimental pulmonary fibrosis both in an *in vivo* mouse model of the disorder and *in vitro.* Finally, we found that the anti-pulmonary fibrotic activity of SAB was mediated by inhibition of Smad-dependent and -independent TGF-β signaling pathways.

## Results

### SM is the most important component of Yiqihuoxue formula

**Figure 1:**
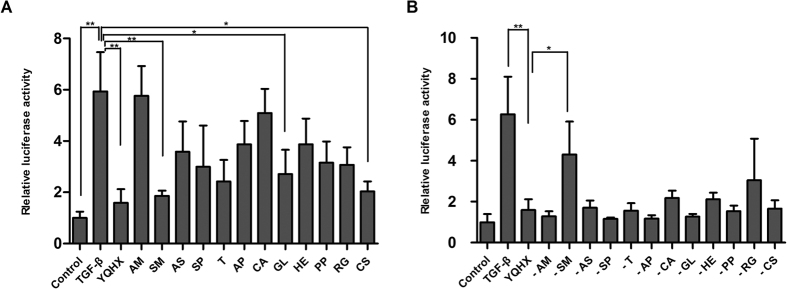
SM was the Yiqihuoxue formula component that most strongly inhibited the TGF-β pathway. (**A**) Relative activity of SBE, measured as luciferase activity, in NIH/3T3 fibroblasts treated with different components of the Yiqihuoxue formula. (**B**) Relative activity of SBE in NIH/3T3 fibroblasts treated with Yiqihuoxue formula from which single components had been removed. Data is presented as mean ± SD of three samples. Significant differences (as determined using one-way ANOVA test) are indicated by asterisks: *P < 0.05; **P < 0.01. YQHX, Yiqihuoxue formula; AM, *Astragalus membranaceus*; SM, *Salviae Miltiorrhiza*; AS, *Angelica sinensis*; SP, *Semen Persicae*; T, *Tuyuan*; AP, *Agkistrodon piscivorus*; CA, *Centella Asiatica*; GL, *Ganoderma Lucidum*; HE, *Herba epimedii*; PP, *Poria peel*; RG, *Radix Glycyrrhizae*; CS, *Caulis Spatholobi*.

Our previous study revealed that Yiqihuoxue formula which is composed of multiple components had a curative effect on fibrosis via down-regulating the Smad-dependent TGF-β pathway^15^. To determine which component in the Yiqihuoxue formula was most important in the inhibition of the Smad-dependent TGF-β pathway, we performed luciferase reporter gene assays using pGL3-SBE_4_-Luc plasmids, which report binding to SBE. Luciferase reporter gene assay showed that TGF-β markedly increased luciferase activity in NIH/3T3 fibroblasts transfected with pGL3-SBE_4_-Luc plasmids. As expected, treatment with the Yiqihuoxue formula significantly downregulated SBE reporter activity. Of the multiple Yiqihuoxue formula components tested individually, SM, GL, and CS significantly inhibited SBE activity (Fig. 1A). Furthermore, when SM was removed from the Yiqihuoxue formula, its inhibitory effect on SBE activity was attenuated significantly (Fig. 1B). Based on the present data, we considered that SM was the most important anti-fibrotic component in Yiqihuoxue formula by down regulation of the TGF-β signaling pathway.

### SAB ameliorated pulmonary fibrosis in bleomycin-induced mouse model

**Figure 2:**
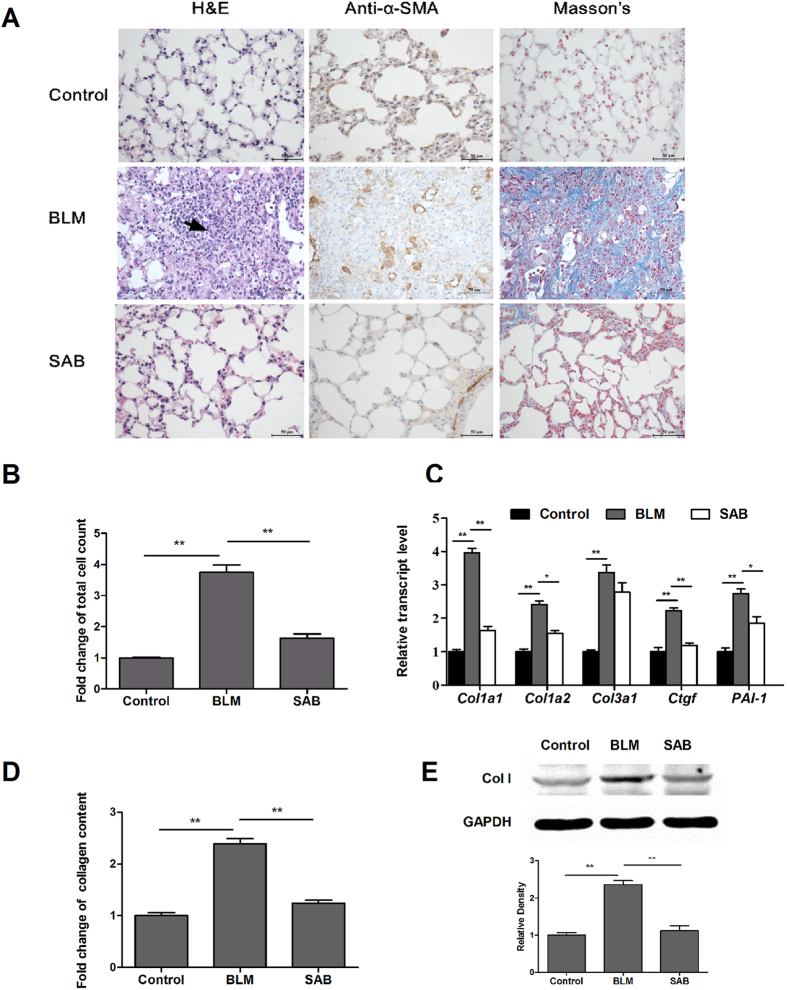
SAB alleviated bleomycin-induced lung fibrosis in mice in the prevention group. (**A**) Sections were stained with hematoxylin and eosin (H&E) and Masson’s trichrome, and immunohistochemistry was performed with an anti-α-SMA antibody. The arrow indicates infiltrated inflammatory cells. Bars = 50 μm. (**B**) Cell counts of lymphocytes from bronchoalveolar lavage fluid (BALF). (**C**) Relative transcript levels of *Col1a1*, *Col1a2*, *Col3a1*, *Ctgf*, and *PAI-1* were determined by real-time RT-PCR. (**D**) Collagen content in the lung tissue from experimental mice was measured using a Sircol collagen kit as described in Materials and Methods. (**E**) The protein level of collagen type I was determined by western blot (1) and analyzed by densitometry (2). Data are presented as means ± SEM of the group and compared by Student’s t test (n = 6); *P < 0.05, **P < 0.01.

SAB is the major water-soluble ingredient extracted from SM, thus we studied the effect of SAB on pulmonary fibrosis *in vivo*. In the prevention group, administration of SAB which began third day before bleomycin instillation resulted in strong amelioration of bleomycin-induced injury and a sharp reduction in the number of infiltrated inflammatory cells, determined by H&E staining of lung tissue and counting of cells in the bronchoalveolar lavage fluid (BALF) (Fig. 2). Furthermore, H&E and Masson’s trichrome staining revealed that bleomycin instillation led to a disruption of the normal lung architecture and an elevation in collagen content in the lungs. However, mice injected with SAB presented substantially less architecture disruption and collagen deposition (Fig. 2A). SAB reduced the increased number of α-SMA-expressing cells in the lung alveoli of mice with bleomycin-induced injury (Fig. 2A). The level of fibrosis was determined by expression profiling of collagen and the pro-fibrotic genes *Col1a1*, *Col1a2*, *Col3a1*, *Ctgf*, and *PAI-1*. Expression of these genes was increased in mice instilled with bleomycin compared to control mice. SAB administration significantly reduced expression of *Col1a1* and *Col1a2* in bleomycin-instilled mice (Fig. 2C). Expression of *Ctgf* and *PAI-1* in these mice was also significantly downregulated by SAB. To more quantitatively examine the level of collagen accumulation, which represents the degree of fibrosis, total lung collagen levels were determined by Sircol collagen dye binding assay. Bleomycin instillation markedly increased the collagen content of lung by 2.5-fold (Fig. 2D). After SAB administration, collagen accumulation in the lungs of bleomycin-exposed mice was decreased by 47.9%. Western blot analysis further indicated that SAB decreased bleomycin-induced expression of type I collagen (Fig. 2E). Moreover, SAB treatment also significantly attenuated the pulmonary fibrosis in bleomycin-instilled mice in the Therapy group, in which group SAB treatment was administered 10 days post-bleomycin instillation (Supplementary Fig. S2).

### SAB suppressed TGF-β-induced myofibroblastic differentiation in lung fibroblasts

**Figure 3:**
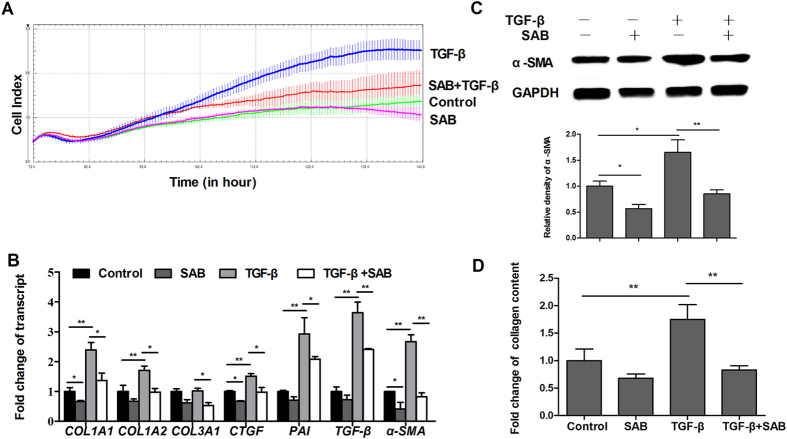
The anti-fibrotic role of SAB in cultured MRC-5 fibroblasts stimulated with TGF-β. (**A**) Growth curves (measured as Cell Indices over time) of MRC-5 fibroblasts treated with vehicle or with TGF-β, SAB, or both, were measured with the xCELLigence system. (**B**) Relative transcript levels of *COL1A1*, *COL1A2*, *COL3A1*, *CTGF*, *PAI-1*, *TGF-β*, and *α-SMA* in the different treatment groups. (**C**) The protein level of α-SMA in the different treatment groups was determined by immunoblot and analyzed by densitometry. (**D**) The total soluble collagen in cell culture supernatants was quantified using the Sircol collagen assay kit. Data is presented as mean ± SD of three samples and compared by Student’s t test or one-way ANOVA test; *P < 0.05, **P < 0.01.

TGF-β plays a key role in driving the differentiation of fibroblasts to myofibroblasts, which helps to promote cell proliferation and ECM deposition. Cell detection by the xCELLigence system showed that TGF-β stimulated the proliferation of MRC-5 fibroblasts, whereas treatment with SAB (50 μg/ml) inhibited this proliferation with no obvious toxicity (Fig. 3A). Real-time RT-PCR showed that TGF-β increased the expression of *COL1A1* and *COL1A2* by 240% and 170%, respectively, in MRC-5 fibroblasts, and that SAB significantly downregulated TGF-β-induced expression of *COL1A1*, *COL1A2*, and *COL3A1* (Fig. 3B). Moreover, TGF-β treatment highly increased the mRNA levels of the pro-fibrotic genes *CTGF*, *PAI-1*, *TGF-β*, and *α-SMA* by 150%, 290%, 360% and 260%, respectively, relative to control. In contrast, SAB substantially downregulated mRNA levels of *CTGF*, *α-SMA* and *PAI-1* (Fig. 3B). Western blot analysis indicated that TGF-β treatment increased protein levels of α-SMA in MRC-5 fibroblasts, whereas SAB decreased its expression significantly (Fig. 3C). Consistent with the results of real-time RT-PCR, stimulation of MRC-5 fibroblasts with TGF-β increased the release of collagen protein, while addition of SAB reduced collagen release (Fig. 3D). As shown in Fig. 3, the inhibitory effect of SAB on TGF-β-treated MRC-5 cells was stronger than that in cells untreated with TGF-β. SAB also induced downregulation of collagen expression and changes in expression of other fibrotic genes in NIH/3T3 fibroblasts (Supplementary Fig. S3).

**Figure 4:**
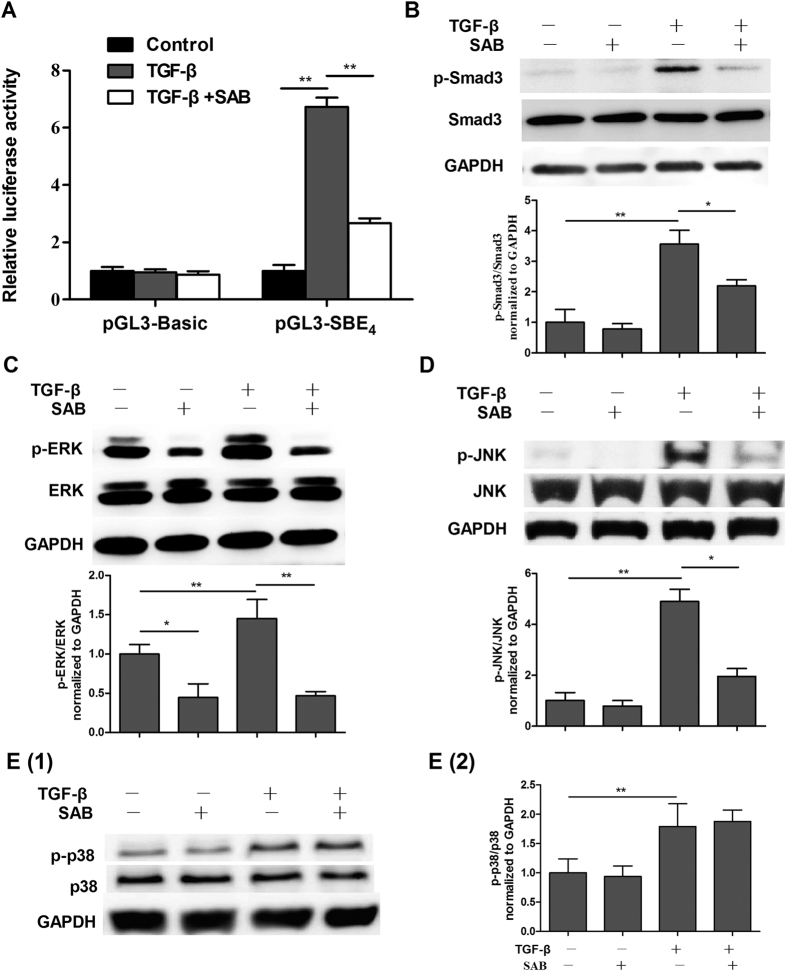
The effects of SAB on the Smad signaling pathway and on the MAPK signaling pathway. (**A**) The effect of SAB on TGF-β-induced SBE_4_ reporter gene activity was measured using a dual-luciferase reporter assay system. (**B–E**) MRC-5 fibroblasts were pretreated with SAB (50 μg/ml) for 24 hours, and then treated with TGF-β (10 ng/ml) for another 30 min. Phosphorylation of Smad3 (**B**), ERK (**C**), JNK (**D**), and p38 (**E**) was determined by western blot and analyzed by densitometry. Data are presented as mean ± SD of three samples and compared by Student’s t test or one-way ANOVA test; *P < 0.05, **P < 0.01.

The Smad pathway is the primary signaling pathway downstream of TGF-β. We investigated the effect of SAB on the Smad-dependent pathway in NIH/3T3 fibroblasts by using the luciferase reporter vector pGL3-SBE_4_-Luc. As expected, TGF-β markedly increased SBE reporter activity by 6.7-fold in fibroblasts transfected with pGL3-SBE_4_-Luc plasmids. Luciferase activity was specifically downregulated in the presence of SAB (Fig. 4A), and luciferase activity in NIH/3T3 fibroblasts transfected with pGL3-Basic control plasmids remained unchanged after TGF-β and SAB stimulation. These results indicate that SAB inhibits the Smad-dependent pathway in fibroblasts. Western blot assays revealed that SAB decreased TGF-β-induced expression of phosphorylated Smad3 protein in MRC-5 fibroblasts (Fig. 4B). As for the Smad-independent pathway, Fig. 4 shows that TGF-β stimulated phosphorylation of ERK1/2, p38, and JNK in MRC-5 fibroblasts. In contrast, SAB substantially inhibited phosphorylation of ERK1/2 and JNK, but had no effect on p38 phosphorylation. These results support the notion that SAB plays an anti-fibrotic role in MRC-5 fibroblasts by inhibiting TGF-β-induced phosphorylation of Smad3, ERK1/2, and JNK proteins, and by affecting both Smad-dependent pathways and the Smad-independent ERK/MAPK and JNK/MAPK signaling pathways.

### Inhibition of TGF-β-induced EMT in A549 cells by SAB

**Figure 5:**
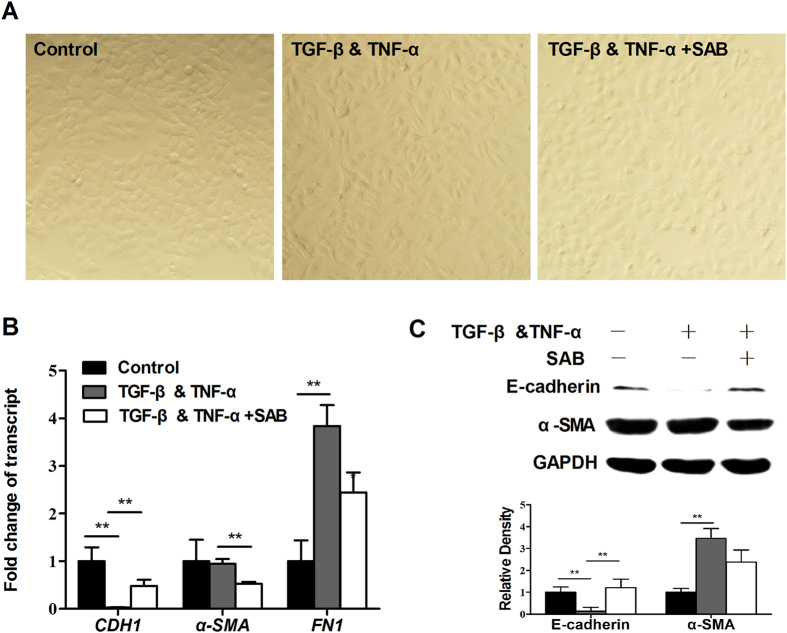
Inhibition of TGF-β-induced ΕΜΤ by SAB in A549 cells. A549 cells were incubated with TGF-β (10 ng/ml) and TNF-α (10 ng/ml) in the absence or presence of SAB (50 μg/ml) for 48 h. (**A**) Morphological changes were imaged using Olympus IX70 microscope (shown at 200× magnification). (**B**) Expression of *CDH1*, *FN1* and *α-SMA* was measured by real-time RT-PCR. (**C**) The protein levels of E-cadherin and α-SMA were determined by immunoblot and analyzed by densitometry. Data are presented as mean ± SD of three samples and compared with Student’s t test; *P < 0.05, **P < 0.01.

To examine SAB’s effect on TGF-β-induced EMT, human alveolar epithelium-derived A549 cells were stimulated with TGF-β and TNF-α (TGF-β & TNF-α). As shown in Fig. 5A, untreated A549 cells exhibited a cobblestone-like epithelial morphology. In contrast, cells grown in the presence of TGF-β & TNF-α displayed a stellate and elongated fibroblast-like morphology, representing morphological changes associated with EMT. Addition of SAB substantially recovered the effects of morphological changes induced by TGF-β & TNF-α (Fig. 5A). Real-time RT-PCR showed that TGF-β & TNF-α treatment substantially downregulated expression of the epithelial phenotypic marker *CDH1*, and upregulated expression of the mesenchymal marker *FN1* compared with the control group (Fig. 5B). In cells treated with SAB, *CDH1* expression was significantly increased and *FN1* expression was significantly repressed compared to cells in the TGF-β & TNF-α group. Western blot analysis further indicated that SAB attenuated TGF-β & TNF-α-induced inhibition of E-cadherin (encoded by *CDH1*) expression (Fig. 5C). These results indicate that SAB inhibits the process of EMT in fibrotic lung cells.

**Figure 6:**
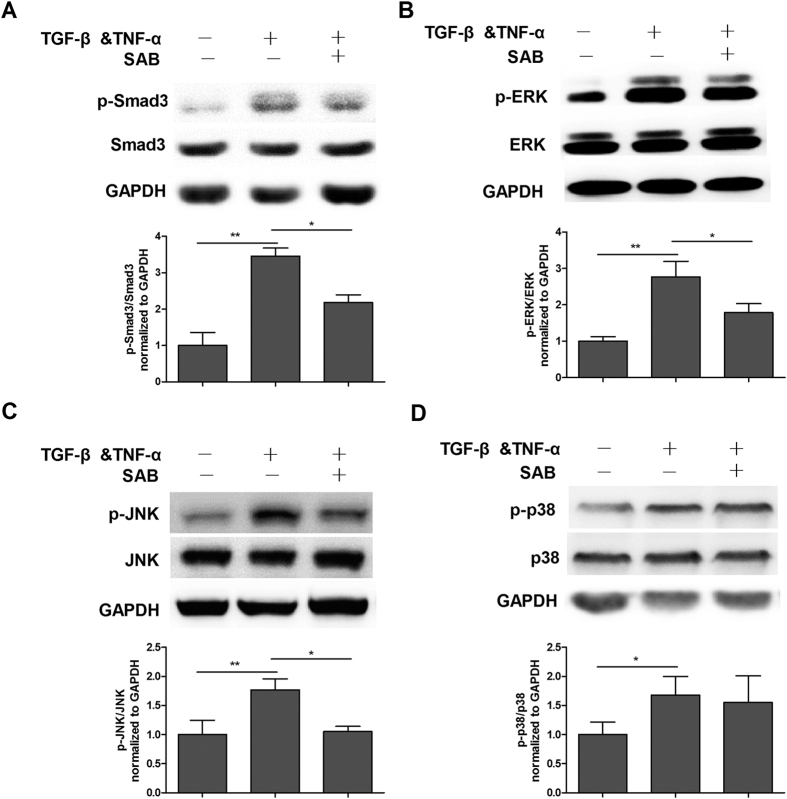
The effects of SAB on the Smad signaling pathway and the MAPK signaling pathway in A549 cells. A549 cells were pretreated with SAB (50 μg/ml) for 24 h, and then treated with TGF-β & TNF-α for another 30 min. Phosphorylation of Smad3 (**A**), ERK (**B**), JNK (**C**) and p38 (**D**) was determined by western blot and analyzed by densitometry. Data are presented as mean ± SD of three samples and compared by Student’s t-test; *P < 0.05, **P < 0.01.

To explore the mechanisms by which SAB inhibited EMT, we examined the influence of SAB on TGF-β-Smad signaling, which is known to play a major role in TGF-β-induced EMT. Expression of phosphorylated Smad3 was significantly increased after incubation with TGF-β & TNF-α. In contrast, SAB treatment substantially attenuated the TGF-β & TNF-α-induced upregulation of p-Smad3 (Fig. 6A). Treatment with TGF-β & TNF-α also resulted in a remarkable increase in p-ERK1/2, p-p38, and p-JNK expression, while SAB suppressed ERK1/2 and JNK phosphorylation (Fig. 6). However, SAB had no effect on p38 phosphorylation, consistent with the results observed in MRC-5 fibroblasts.

## Discussion

Our findings demonstrated that SM is the most important anti-fibrotic component of the Yiqihuoxue formula. They further revealed that SAB, the major water-soluble ingredient of SM, effectively alleviated experimental pulmonary fibrosis both *in vivo* and *in vitro*. This anti-fibrotic effect was mediated, at least in part, by inhibition of the TGF-β signal transduction pathway.

The Yiqihuoxue formula, which is composed of 12 components, has a therapeutic effect on scleroderma and scleroderma-related lung fibrosis^17^,^18^. Our previous study showed that the therapeutic effects of the Yiqihuoxue formula on scleroderma were mediated by inhibition of the TGF-β pathway^15^. We performed this study to determine which ingredients of the Yiqihuoxue formula were important in mediating its anti-fibrotic effect, and to further examine its effect on pulmonary fibrosis. We found that SM was the most important anti-fibrotic component of the Yiqihuoxue formula in down-regulation of SBE activity (Fig. 1).

SM has been widely used in China and, to a lesser extent, in Japan, the United States, and European countries, for the treatment of cardiovascular and cerebrovascular diseases^19^. The Fufang Danshen Dripping Pill, a dosage form of SM, became the first TCM product approved for phase II and III clinical trials by the Food and Drug Administration (FDA) in 1997 (IND No. 56956)^19^. SAB is the predominant ingredient yielded when SM is processed traditionally by extraction with water. SAB is the primary mediator of the cellular functions of SM, especially its antioxidative and free radical scavenging effects^20^. As expected, SAB significantly down-regulated the SBE reporter activity which could also be suppressed by Yiqihuoxue formula in NIH/3T3 fibroblasts (Fig. 4). It has been reported that SAB could effectively reverse liver fibrosis in patients suffering from chronic hepatitis B^16^. Furthermore, SAB could possibly facilitate the repair of tubular epithelial structures and the regression of renal fibrosis in injured kidneys^21^. Therefore, we carried out further *in vivo* and *in vitro* studies to examine the effect as well as the mechanism of SAB on pulmonary fibrosis.

A mouse model of bleomycin-induced pulmonary fibrosis was used to assess the effects of SAB. Bleomycin-induced lung injury has an early inflammatory phase characterized by leukocyte infiltration and a subsequent fibrotic phase^22^. In the present study, we demonstrated that long-term SAB administration attenuated bleomycin-induced inflammatory cell infiltration and collagen-mediated fibrosis in mouse lung in the prevention group. Specifically, SAB substantially reduced the deposition of collagen and the expression of several fibrogenic cytokines (Fig. 2). Furthermore, we found that SAB treatment administered 10 days post-bleomycin instillation suppressed bleomycin-induced pulmonary fibrosis. Our results also suggest that SAB can attenuate bleomycin-induced pulmonary fibrosis by decreasing ECM deposition. Based on the above results, we speculate that SAB attenuated bleomycin-induced lung fibrosis by inhibiting both the inflammation phase and the fibrosis phase. Mounting evidence suggests that SAB is capable of preventing the development of cancer, and that anti-inflammatory mechanisms of SAB could involve modulation of cytokines, the COX-2/PGE-2 pathway, NF-κB, TNF-α, and MMPs^23^. Furthermore, SAB attenuates LPS-induced pulmonary microcirculatory disturbance, including the increase in leukocyte adhesion and albumin leakage in rats^24^. In future study, we will further investigate the anti-inflammatory role of SAB in pulmonary fibrosis.

The increased proliferation of resident fibroblasts, fibroblast-myofibroblast transdifferentiation, and accumulation of extracellular matrix are critical in the development of pulmonary fibrosis^25^. Our *in vitro* findings showed that SAB significantly inhibited the proliferation of MRC-5 lung fibroblasts and downregulated collagen expression at both the transcriptional and translational levels. Moreover, the inhibitory effects of SAB on proliferation and differentiation were greater in MRC-5 cells treated with TGF-β than in untreated MRC-5 cells. In addition, SAB inhibited TGF-β-induced mRNA expression of the fibrotic genes *CTGF*, *PAI-1*, *TGF-β*, and *α-SMA* in MRC-5 fibroblasts. As a gene downstream of the TGF-β signaling pathway, CTGF is associated with potent and persistent fibrotic changes^26^. Downregulation of CTGF has already been employed as a strategy by which to treat fibrotic conditions^27^. PAI-1, a member of the serine protease superfamily, can suppress collagen dissolution and promote its accumulation^28^. Bauman *et al*.^29^ reported that PAI-1 deficiency protected lungs from bleomycin-induced lung fibrosis^29^. Fibrosis is mediated by a specialized, activated form of fibroblast called a myofibroblast. Myofibroblasts are the main producers of the ECM and are characterized by their high expression of the α-SMA protein. In the present study, SAB reversed the mRNA expression of *CTGF*, *α-SMA*, and *PAI-1* induced by TGF-β. This finding provides further support for the anti-fibrotic role of SAB.

Our data also showed that SAB significantly blocked the TGF-β-mediated downregulation of the epithelial marker E-cadherin and the TGF-β-mediated upregulation of the mesenchymal cell markers FN1 and α-SMA. SAB also attenuated TGF-β-mediated changes in epithelial morphology in A549 cells. E-cadherin is an adherent junction protein that is functionally linked to the generation of a polarized epithelial phenotype^30^. The upregulation of E-cadherin by SAB may explain the ability of SAB to prevent TGF-β & TNF-α-induced morphological changes associated with the EMT. Inhibition of the EMT by SAB is further evidence of its anti-fibrotic role.

The TGF-β signaling pathway, which can be Smad-dependent or -independent, has been considered the most important pathway involved in the activation of fibroblasts, the EMT, and the pathogenesis of lung fibrosis^31^. Our data showed that SAB could reduce TGF-β-induced phosphorylation of Smad3 protein and downregulation of the Smad transactivation reporter in fibroblasts. SBE has been found in the promoters of many TGF-β-induced genes, including CTGF, α-SMA and PAI-1. Moreover, our previous data demonstrated that SAB inhibited the transcription of these TGF-β-induced genes in MRC-5 fibroblasts. This inhibition of gene transcription may have occurred through SAB attenuating the phosphorylation of Smad3, thereby interfering with its binding to SBE and resulting in the reduction of gene transcription. In the A549 cells, SAB treatment also significantly inhibited the TGF-β-induced upregulation of p-Smad3. SAB more strongly inhibits Smad3 phosphorylation in A549 cells than in MRC-5 cells; further study is required to determine the specific mechanisms mediating this difference. Smad-independent signals like the MAPK signaling pathways have also been suggested to play a role in TGF-β-mediated pro-fibrotic effects, including EMT induction^11^,^32^. In this study, we demonstrated that SAB significantly inhibited the phosphorylation of both ERK and JNK, but not p38, in TGF-β-stimulated MRC-5 fibroblasts and A549 cells. Previous findings have shown that the ERK kinase cascade phosphorylates Smad and modifies Smad activity, and ERK is involved in fibroblast proliferation in addition to the induction of some profibrotic genes^33^. Excessive activation of JNK is also relevant to pulmonary fibrosis. Hashimoto *et al*. have demonstrated that the JNK signaling pathway is important in the differentiation of fibroblasts to myofibroblasts in human lungs^34^. Collectively, these results showed that SAB plays an anti-fibrotic role via regulation of the TGF-β/Smad signaling pathway and the MAPK signaling pathway. However, the exact extent by which each pathway is downregulated by SAB and contributes to its effects is difficult to estimate because there is complicated cross-talk between the two pathways. Hence, more in-depth studies of their roles are still needed.

In conclusion, our study is the first to demonstrate that treatment with SAB remarkably alleviated bleomycin-induced pulmonary fibrosis. Furthermore, our *in vitro* experiments revealed that SAB suppressed TGF-β-induced myofibroblastic differentiation in MRC-5 fibroblasts and TGF-β-mediated EMT in A549 cells by suppressing the TGF-β pathway. These findings raise the possibility that SAB, via inhibiting the TGF-β signaling pathway, is an effective drug for the treatment of pulmonary fibrosis.

## Methods

### Cell culture and reagents

Human embryonic lung fibroblast (MRC-5) cell line, human type II alveolar epithelial cell line (A549) and mouse embryonic fibroblasts cell line (NIH/3T3) were obtained from the Shanghai Institute of Cell Biology, Chinese Academy of Sciences (Shanghai, China). MRC-5 fibroblasts were cultured in Minimum Essential Medium (MEM) medium supplemented with 10% fetal bovine serum (FBS) at 37 °C in a 5% CO_2_ humidified incubator. A549 cells and mouse NIH/3T3 cells were cultured in Dulbecco’s Modified Eagle Medium (DMEM) supplemented with 10% FBS. SAB (purity >90%) was obtained from Shanghai Lvgu Pharmaceutical Co. Ltd. It was freshly prepared in phosphate buffer solution (PBS) before use. Recombinant TNF-α and recombinant TGF-β was purchased from R&D Systems (Minneapolis, MN, USA). Antibodies to E-cadherin and α-SMA were purchased from Abcam (New Territories, Hong Kong). Antibodies to Smad3, p-Smad3, p-ERK, ERK, p-P38 and p-JNK were purchased from Cell Signaling Technology (Boston, USA). Collagen type I antibody was from Millipore (Billerica, MA, USA).

### Luciferase reporter gene assay

To investigate the effect of Yiqihuoxue formula or SAB on the Smad-dependent TGF-β pathway in fibroblasts, the luciferase reporter vector pGL3-SBE_4_-Luc was used. The Yiqihuoxue formula was prepared as previously described^15^. pGL3-SBE_4_-Luc was a gift from Dr. Kiyoshi Higashi (Sumitomo Chemical Co., Ltd., Osaka, Japan), and consists of four short tandem repeats of the SBE oligonucleotide (GTCTAGAC) inserted into the pGL3 Basic vector^35^. pGL3-Basic or pGL3-SBE_4_-Luc reporter plasmids were co-transfected with pRL-SV40 plasmids into NIH/3T3 fibroblasts using Lipofectamine 2000 (Invitrogen, Carlsbad, CA, USA) according to the manufacturer’s protocol. Six hours post transfection, 1% FBS DMEM containing 10 ng/ml TGF-β alone or together with 10 mg/ml Yiqihuoxue formula or 50 μg/ml SAB was added to each well. Twenty-four hours later, luciferase activity assay was performed according to the manufacturer’s protocol for the Dual-luciferase reporter assay system (Promega, Madison, WI, USA) with a GloMax 20/20 Luminometer (Promega). Transcriptional activities of reporter constructs were normalized against those of cotransfected pRL-SV40. Each value represents mean ± standard deviation (SD) of three independent experiments.

### Bleomycin-induced pulmonary fibrosis mouse model

Experiments using animals were approved by the Institutional Animal Care and Use Committee of Fudan University, and the methods were carried out in accordance with the approved guidelines^15^. C57BL/6 mice were purchased from the Fudan University Animal Center. C57BL/6 mice aged 6–7 weeks were used for study of pulmonary fibrosis. Mice were randomly divided into two groups: a Prevention group and a Therapy group. There were three subgroups in each group as follows: a Control group, a Bleomycin (BLM) group, and a SAB group. Bleomycin (50 μl, 3.5 units/kg, diluted in sterile saline) was instilled intratracheally (IT) into mice in the BLM and SAB groups, while mice in the Control group received equal volumes of saline. Mice in the SAB subgroups were given daily intraperitoneal (IP) injections of SAB (10 mg/kg body weight). For the SAB subgroup mice within the Prevention group, injections began the third day before bleomycin instillation, whereas for those within the Therapy group, injections began on the tenth day after bleomycin instillation. All mice were killed 3 weeks after bleomycin administration. Lung tissues were either flash frozen and then stored in liquid nitrogen for further analysis or perfused and fixed in 10% formalin for at least 24 h at room temperature for immunohistochemical analysis.

### Histological analysis

Sections were stained with hematoxylin and eosin (H&E) to evaluate the severity of lung inflammation and lung fibrosis. Masson Trichrome staining was performed to assess the degree of fibrosis. Immunohistochemical analysis of the lung tissue samples was performed following standard procedures. Briefly, endogenous peroxidase activity was blocked by immersion of deparaffinized sections in 3% H_2_O_2_ in methanol for 30 min. Antigen retrieval was performed by steaming slides in 0.01 M citrate buffer (pH 6.0) for 30 min. Slides were blocked with 1% bovine serum albumin for 30 min at room temperature and subsequently incubated at 4 °C overnight with antibodies against α-smooth muscle actin (α-SMA). Slides were then incubated at 37 °C for 1 h with horseradish peroxidase-conjugated goat anti-rabbit IgG secondary antibody. Between each incubation, sections were washed three times with PBS. Sections were developed with 3, 3-diaminobenzidine tetrahydrochloride and hydrogen peroxide and subsequently counterstained with hematoxylin. The sections were imaged using a Nikon Eclipse 80i microscope (Nikon, Badhoevedorp, the Netherlands) and assessed at 200× magnification.

### Collagen measurements

Total soluble collagen in cell culture supernatants and lung samples was quantified using the Sircol collagen assay (Biocolor, Belfast, UK) according to the manufacturer’s protocol. The amount of collagen protein in lung samples was normalized to the total amount of protein as determined using a BCA Protein Assay kit (Beyotime, Nanjing, China).

### Proliferation assay

For time-dependent cell response profiling, 50 μl of media was added to the E-Plates 16 (Roche, East Sussex, UK) to obtain background readings. 50 μl of suspended cells were then seeded onto E-plates 16 and cell growth was measured with the xCELLigence system (Roche) as previously reported^36^. After 24 h, the cells were treated with SAB and/or TGF-β. Every ten minutes, the cell density was measured as the cell index, a unit indicating the percentage of the well occupied by cells.

### RNA Isolation, cDNA Synthesis, and Real-time RT-PCR

Total RNA was extracted from cells using TRIzol reagent according to the manufacturer’s instructions (Invitrogen). Reverse transcription was performed using a High Capacity cDNA Reverse Transcription Kit (Applied Biosystems, Foster City, CA, USA) according to the manufacturer’s protocol. Real-time PCR samples were prepared with SYBR Premix Ex Taq (TakaRa Biotech, Tokyo, Japan) and real-time PCR was performed with an ABI Prism 7900 Detector System (Applied Biosystems). The housekeeping gene *GADPH* was used as an internal control. The Real-time RT-PCR primers are listed in Supplementary Table S1. The data obtained from the assays were analyzed with SDS 2.3 software (Applied Biosystems).

### Western blot analysis

The cellular lysates extracted from cells were used for protein assays. Protein concentration was detected by a spectrophotometer using a BCA protein assay kit. Equal amounts of protein were subjected to SDS-PAGE on a 10% poly-acrylamide gel and transferred to a polyvinylidene difluoride membrane (Millipore). The membrane with blotted protein was blocked for 1 h at room temperature with blocking buffer containing 5% BSA, followed by incubation with antibodies overnight at 4 °C. GAPDH was used as an internal control. Then, after three 10 min washes with TBST, the membrane was incubated for 1 h at room temperature with secondary horseradish peroxidase-conjugated goat anti-rabbit, or anti-mouse IgG. The protein bands were visualized with ECL solution.

### Statistical analysis

All experiments were performed in triplicate. The statistical analyses were performed with the SPSS 16.0 software, a P value of less than 0.05 was considered significant. Either an independent two group t-test or one-way ANOVA test with post hoc Dunnett’s multiple comparison test were used for the evaluation of significance between different groups.

## Additional Information

**How to cite this article**: Liu, Q. *et al*. Salvianolic Acid B Attenuates Experimental Pulmonary Fibrosis through Inhibition of the TGF-β Signaling Pathway. *Sci. Rep.* **6**, 27610; doi: 10.1038/srep27610 (2016).

## Supplementary Material

Supplementary Information
