# Supplementary material for: Salvianolic Acid B Attenuates Experimental Pulmonary Fibrosis through Inhibition of the TGF-β Signaling Pathway
Source: Sci Rep. 2016 Jun 9;6:27610. doi: 10.1038/srep27610 (PMC4899783; doi:10.1038/srep27610)
Supplement: Supplementary Information [file srep27610-s1.pdf]

Salvianolic Acid B Attenuates Experimental Pulmonary Fibrosis through Inhibition of the TGF-β Signaling Pathway

Qingmei Liu[1], Haiyan Chu[1], Yanyun Ma[1], Ting Wu[1], Feng Qian[1], Xian Ren[2], Wenzhen Tu[3,6], Xiaodong Zhou[4], Li Jin[1], Wenyu Wu[5,6,*] & Jiucun Wang[1,6,*]

[1]State Key Laboratory of Genetic Engineering and Ministry of Education Key Laboratory of Contemporary Anthropology, Collaborative Innovation Center for Genetics and Development, School of Life Sciences and Institutes of Biomedical Sciences, Fudan University, Shanghai, China.

[2]Shanghai Green Valley Pharmaceutical Co., Ltd, Shanghai, China.

[3]Department of Dermatology, Shanghai TCM-integrated Hospital, Shanghai, China.

[4]Division of Rheumatology, University of Texas-Houston Health Science Center, Houston, USA.

[5]Division of Dermatology, Huashan Hospital, Fudan University, Shanghai, China.

[6]Institute of Rheumatology, Immunology and Allergy, Fudan University, Shanghai, China.

* Corresponding author. Correspondence and requests for materials should be addressed to W. W. (wuwenyu@medmail.com.cn) or J. W. (email: jcwang@fudan.edu.cn)

Supplementary Figure S1

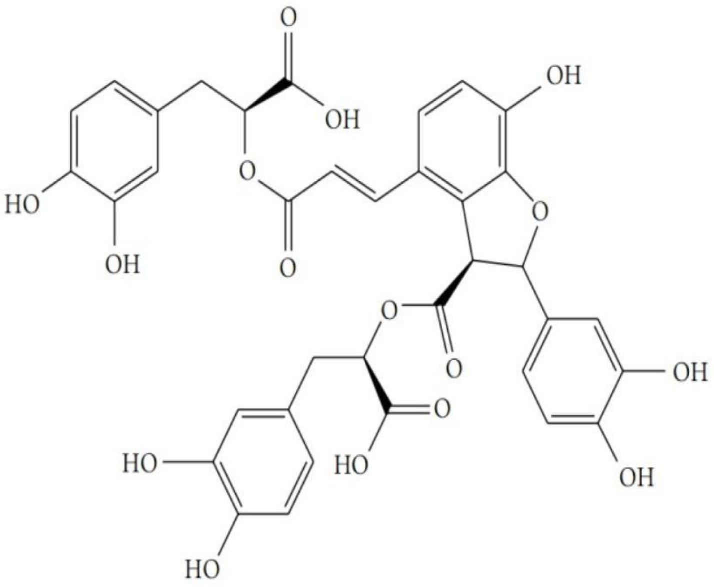

Supplementary Figure S1: The chemical structure of Salvianolic acid B.

Supplementary Figure S2

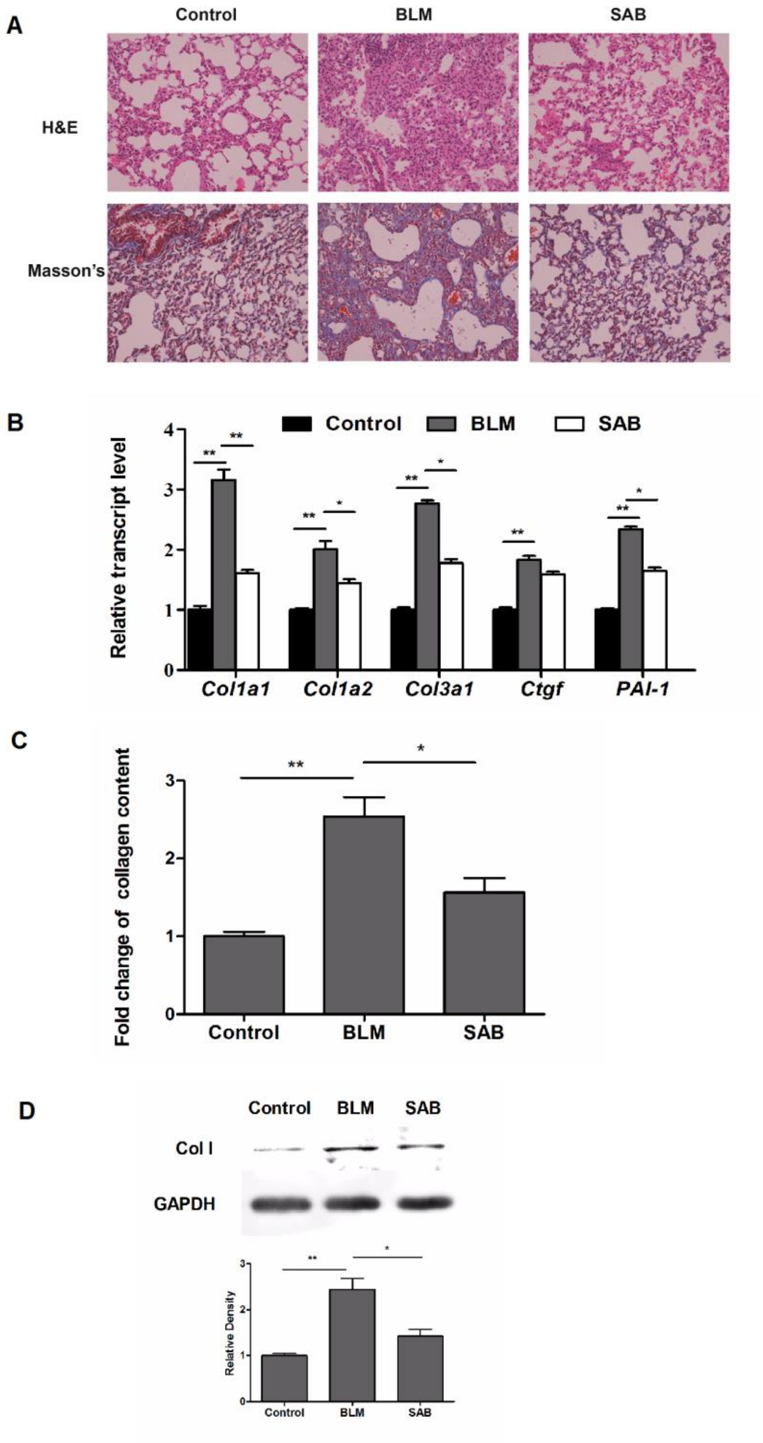

Supplementary Figure S2: SAB alleviated bleomycin-induced lung fibrosis in mice in the Therapy group. (**A**) Sections were stained with hematoxylin and eosin (H&E) and Masson's trichrome, and the sections were assessed at 200× magnification. (**B**) Relative transcript levels of *Col1a1*, *Col1a2*, *Col3a1*, *Ctgf*, and *PAI-1* were

determined by real-time RT-PCR. (**C**) Collagen content in the lung tissue from experimental mice was measured using a Sircol collagen kit as described in Materials and Methods. (**D**) The protein level of collagen type I was determined by western blot and analyzed by densitometry. Data are presented as means ± SEM of the group and compared by Student's t test ($n = 7$); *$P < 0.05$, **$P < 0.01$.

Supplementary Figure S3

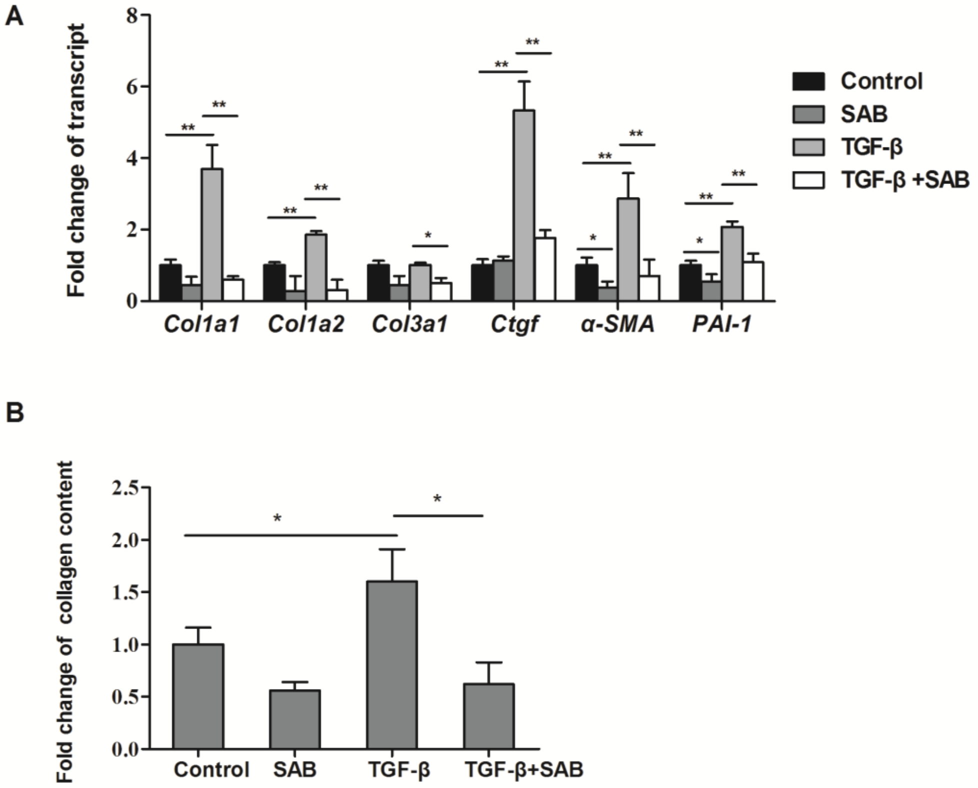

Supplementary Figure S3: The anti-fibrosis role of SAB in NIH/3T3 fibroblasts stimulated with TGF-β. (**A**) Relative transcript levels of *Col1a1*, *Col1a2*, *Col3a1*, *Ctgf*, *a-SMA* and *PAI-1* in different treated groups. (**B**) The total soluble collagen in cell culture supernatants was quantified using the Sircol collagen assay kit. Data was means ± SD of three samples and compared by Student's t test; **P* < 0.05, ***P* < 0.01.

Supplementary Table S1

Supplementary Table S1. Real-time RT-PCR Primer Sequences.

Primer Name	Species	Sequence 5' to 3'
Col1a1-F	Mouse	GGTCCACAAGGTTTCCAAGG
Col1a1-R	Mouse	GCTGTTCCAGGCAATCCAC
Col1a2-F	Mouse	GGACCCGTTGGCAAAGATG
Col1a2-R	Mouse	CACCAGGAGGACCAGGAG
Col3a1-F	Mouse	GAGGAAACAGAGGTGAAAGAGG
Col3a1-R	Mouse	CAGCAATGGCAGCAGCAC
*Ctg*f-F	Mouse	AAGGACCGCACAGCAGTTGG
Ctgf-F	Mouse	AGGCAGTTGGCTCGCATCATAG
PAI-1-R	Mouse	GACTATGGTGAAACAGGTGGACT
PAI-1-R	Mouse	TGCTGGCCTCTAAGAAAGGAG
Gapdh-F	Mouse	AACTCCCACTCTTCCACCTTCG
*Gapd*h-R	Mouse	TCCACCACCCTGTTGCTGTAG
COL1A1-F	Human	CATCTGGTGGTGAGACTTGC
COL1A1-R	Human	TCCTGGTTTCTCCTTTGG
COL1A2-F	Human	AAGGTCATGCTGGTCTTGCT
COL1A2-R	Human	GACCCTGTTCACCTTTTCCA
COL3A1-F	Human	GTCCCAGCGGTTCTCCA
COL3A1-R	Human	CCCCGTGCTCCAGTGAT
CTGF-F	Human	GGAAATGCTGCGAGGAGTGG
CTGF-R	Human	GGCTCTAATCATAGTTGGGTCTGG
PAI-1-F	Human	AGTGGACTTTTCAGAGGTGGAG
PAI-1-R	Human	GCCGTTGAAGTAGAGGGCATT
TGF-β-F	Human	AGCAACAATTCCTGGCGATAC
TGF-β-R	Human	CTAAGGCGAAAGCCCTCAAT
α-SMA-F	Human	GAGCGTGGCTATTCCTTCGT
α-SMA-R	Human	GCCCATCAGGCAACTCGTAA
CDH1-F	Human	CGAGAGCTACACGTTCACGG

CDH1-R	Human	GGGTGTCGAGGGAAAAATAGG
FN1-F	Human	CTGAATCTGTGACCGAAAT
FN1-R	Human	GGTACTGTGGCTCATCTCC
GAPDH-F	Human	GAAGGTGAAGGTCGGAGTC
GAPDH-R	Human	GAAGATGGTGATGGGATTTC
